# Association between traditional Chinese medicine constitution and depression in adolescents: A cross-sectional study

**DOI:** 10.1097/MD.0000000000047310

**Published:** 2026-01-23

**Authors:** Yizhao Hao, Jiahui Yin, Zhe Huang, Yueqi Zhang, Yuanyuan Liu, Jingli Zhang, Xingwei Fang, Zhongli Lan, Bin Luo, Min Lu, Zhangjin Zhang, Jun Chen

**Affiliations:** a Department of Preventive Treatment of Disease, Guangd ong Provincial Hospital of Chinese Medicine, Zhuhai, China; b School of Chinese Medicine, LKS Faculty of Medicine, The University of Hong Kong, Hong Kong SAR, China; c Traditional Chinese Medicine Department, Chaozhou People's Hospital, Chaozhou, China; d Department of Psychiatry, Guangdong Provincial Hospital of Chinese Medicine, Zhuhai, China; e Department of Traditional Therapies, Guangdong Provincial Hospital of Chinese Medicine, Zhuhai, China; f Department of Prevention and Health care, Guangdong Provincial Hospital of Chinese Medicine, Zhuhai, China; g Department of Information, Guangdong Provincial Hospital of Chinese Medicine, Zhuhai, China; h The Second Clinical College, Guangzhou University of Chinese Medicine, Guangzhou, China; i National Institute of TCM Constitution and Preventive Medicine, Beijing University of Chinese Medicine, Beijing, China; j Department of Hospital Office, Guangdong Provincial Hospital of Chinese Medicine, Zhuhai, China; k Department of Chinese Medicine, The University of Hong Kong-Shenzhen Hospital, Shenzhen, China; l Department of Radiology, Guangdong Provincial Hospital of Chinese Medicine, Zhuhai, China; m Department of Radiology, Guangdong Provincial Hospital of Chinese Medicine, Guangzhou, China.

**Keywords:** adolescent, depression, traditional Chinese medicine constitution

## Abstract

Traditional Chinese medicine believes that constitution determines individuals’ susceptibility to different health conditions. Previous studies have confirmed the correlation between traditional Chinese medicine constitution (TCMC) and various physical diseases and psychological disorders. However, there is limited research examining the relationship between TCMC and depression in adolescents. A cross-sectional study was conducted among 5955 adolescents aged 14 to 24 years from a vocational technical school in China. The participants completed self-report questionnaires. The TCMC questionnaire was used to assess participants’ constitutions based on 9 different types: balanced constitution, Qi-stagnation constitution, blood-stasis constitution, Qi-deficiency constitution, Yin-deficiency constitution (YIDC), Yang-deficiency constitution (YADC), Phlegm-dampness constitution (PDC), Damp-heat constitution (DHC) and Inherited special constitution (ISC). Depression was accessed using the Self-rating depression scale (SDS). After accounting for confounding factors, balanced constitution were significantly associated with a lower prevalence of depression (odds ratio [OR] = 0.83, 95% confidence interval [CI] 0.72–0.94; *P* = .004). Meanwhile, Qi-deficiency constitution (OR = 1.68, 95% CI: 1.39–2.03; *P* <.001) and Qi-stagnation constitution (OR = 2.53, 95% CI: 2.00–3.23; *P* <.001) were independently associated with a higher prevalence of depression. Moreover, the associations were also identified by the propensity score matching method. Our findings suggest a significant relationship between TCMC and depression in adolescents. Further research is needed to better understand whether these constitutional factors may contribute to or influence the development of depression during adolescence. Integrating Constitution in Chinese Medicine Questionnaire into clinical assessments may provide valuable insights for identifying at-risk individuals and guiding personalized treatments for depression in adolescents.

## 1. Introduction

Depression is a prevalent psychological symptom often linked to clinical depressive disorders such as major depressive disorder (MDD). Adolescents are particularly prone to experiencing depression due to being in a critical period marked by rapid physical, psychological, and social changes.^[1]^ The prevalence of depression among young individuals has significantly risen over the past decade,^[2]^ prompting public concerns. Understanding the factors related to adolescent depression is highly beneficial as it may contribute to the prevention and treatment of depression. For example, integrating relevant factors associated with depression can offer personalized treatment for adolescent patients with depression.^[3]^ Many physical and psychological characteristics have been identified that are associated with the onset and severity of depression in adolescents.^[4–6]^ In traditional Chinese medicine (TCM) theory, depression is typically caused by disruptions in the balance of Qi and the zang-fu meridians, particularly liver-Qi-stagnation and spleen-Qi deficiency, which correspond closely to the constitution types assessed by the TCM constitution (TCMC). Therefore, we propose that TCMC may be a relevant factor in the onset and severity of adolescent depression.

TCM, with a history dating back 3000 years,^[7]^ remains one of the world’s oldest medical systems and continues to be widely practiced today. TCM believes that the body is interconnected and influenced by the environment and emotions, and that the key to good health is achieving balance and harmony within the body. The TCMC within this framework represents an individual’s unique and dynamic state encompassing physical, physiological, and psychological aspects, shaped by both inherent and acquired factors over the course of life.^[8]^ While generally stable, the TCMC is also subject to evolution and adaptation in response to environmental influences over a person’s lifespan. This constitution not only influences susceptibility to specific diseases but also plays a significant role in disease progression and prognosis.

Traditionally, TCMC can be categorized into 2 main types: balanced constitution (BC), also referred to as normality constitution, and unbalanced/biased constitution. The latter can be further subclassified into various types, such as Yang deficiency, Yin deficiency, Phlegm-dampness, Qi deficiency, damp-heat, and Qi-stagnation.^[9]^ In TCM philosophy, a BC signifies a state of overall health, while individuals with unbalanced constitutions are more susceptible to certain diseases.^[10]^

Several studies have provided evidence on the associations between TCMC and various diseases,^[11]^ including anxiety,^[12]^ cognitive decline,^[13]^ ischemic stroke,^[14]^ dyslipidemia,^[15]^ diabetes mellitus,^[16]^ dysfunction-associated fatty liver disease.^[17]^ Adolescents undergo rapid physiological and psychological changes. TCM offers a personalized framework that categorizes individuals into different constitution types, each reflecting specific patterns of organ–meridian balance and emotional resilience. This personalization naturally aligns with precision mental health, allowing for interventions tailored to each adolescent’s unique TCMC profile. However, the relationship between TCMC and adolescent depression remains unclear, with studies in different youth populations yielding inconsistent results.^[18–20]^ Therefore, this study aimed to explore the link between TCMC and depression in adolescents by conducting a quantitative analysis using an observational, cross-sectional research design.

## 2. Materials and methods

### 2.1. Study design and participants

This was a cross-sectional study carried out between September 2023 and January 2024, involving students enrolled in a vocational technical school located in Zhuhai City. This study was approved by the Ethics Committee of Guangdong Provincial Hospital of Chinese Medicine (approval number: ZF2023-401-01) in accordance with the Declaration of Helsinki (2013 revision). All student participants provided written informed consent. For those under 18, additional parental consent was obtained electronically via official school WeChat groups (a widely used social messaging app in China), where parents received a written explanation of the study and provided digital confirmation.

Data collection was performed using self-administered electronic questionnaires. The system was programmed to prevent missing responses by requiring completion of all items before submission. Prior to questionnaire administration, Yizhao Hao, a clinician from Guangdong Provincial Hospital of Chinese Medicine, provided a briefing session to students to explain the study purpose and clarify potential misunderstandings about the questionnaire content. Written instructions preceding the questionnaire also emphasized the importance of honest responses and assured participants that there were no right or wrong answers. Anonymity and voluntariness were strongly emphasized throughout the process. Students were assured that their responses would remain confidential and would not affect their academic performance or school records. With mobilization by the research team, a total of 5994 complete questionnaires were collected from 7316 students across the school. Incomplete responses were not saved or submitted due to the settings of the electronic questionnaire system. The population of interest in this study is adolescents, so we included students aged 14 to 24 in the primary analysis.^[1,21]^ We excluded 39 students who were over 24 years old, resulting in a final analytic sample of 5955 participants. We also conducted parallel analyses using the full dataset, including those above age 24. Lastly, we conducted additional sensitivity analyses focusing on participants aged 14 to 19 years.

### 2.2. Traditional Chinese medicine constitution

We utilized the CCMQ developed by Professor Wang Qi, which demonstrates favorable reliability and validity.^[22–24]^ The questionnaire comprised 60 items and was categorized into 9 dimensions: BC, Qi-stagnation constitution (QSC), Blood-stasis constitution (BSC), Qi-deficiency constitution (QDC), Yin-deficiency constitution (YIDC), Yang-deficiency constitution (YADC), Phlegm-dampness constitution (PDC), Damp-heat constitution (DHC) and inherited special constitution (ISC). Each item is rated on a 5-point Likert scale, reflecting the frequency or intensity of constitution-related traits or symptoms. The development of the CCMQ followed rigorous methodological standards for scale construction, with the test–retest reliability coefficients ranged from 0.77 to 0.90 and Cronbach α coefficients ranging from 0.70 to 0.82.^[23,24]^

The classification criteria used in this study were based on the national standard issued by the China Association of Chinese Medicine (ZYYXH/T157-2009),^[25]^ which provides standardized thresholds for the interpretation of constitution scores. According to this guideline, individuals with a BC score ≥60 and scores <30 in all other constitution types are classified as having a BC. If a participant scores ≥40 in any of the other 8 biased constitution types, they are classified as having that constitution. The BC is considered the epitome of good health. Individuals identified as having a BC are not simultaneously assigned to any biased constitution type. One individual may exhibit one or more biased constitutions. This classification method has been widely applied in both clinical and population-based studies,^[17,26–28]^ supporting its generalizability and practical value in TCMC research. Although no pilot testing was conducted in this study, we employed a standardized and well-validated instrument with established scoring criteria to ensure methodological consistency and comparability of results.

### 2.3. Self-rating depression scale

Depression was assessed using the Zung self-rating depression scale (SDS). A commonly used tool in mainland China.^[29,30]^ The Chinese version of the questionnaire has demonstrated good reliability and validity.^[31,32]^ The SDS consists of 20 items designed to evaluate depressive symptoms. Participants rate each item based on their feelings over the past week using a 4-point rating scale (1–4), with higher scores indicating more frequent symptoms. Elevated SDS scores reflect increased levels of depression. The sum of the scores from the 20 items constitutes the total score, which is then multiplied by 1.25 to obtain the SDS index score. Individuals scoring <50 indicate no depression, 50–59 are classified as having minimal to mild depression, 60 to 69 are categorized as experiencing moderate-to-marked depression, and those scoring 70 and above are deemed to have severe depression.^[33–35]^ Depression was defined as an SDS score of ≥50 in the present study.^[36]^ In addition, a sensitivity analysis was conducted using an alternative outcome definition, in which an SDS score ≥60 was considered to indicate clinically significant depression.^[37]^

## 3. Statistical analysis

Categorical data were presented as counts and percentages. The chi-square test was utilized for the comparison of categorical data. The continuous variables in this study were all non-normal continuous variables, expressed as median (interquartile range [IQR]). Comparisons between groups were conducted using the Wilcoxon rank sum test. Multiple group comparisons were performed using the Kruskal–Wallis H test, and post hoc analysis between 2 groups was carried out using the Bonferroni method.

Correlation factors were analyzed using univariate and multivariate logistic regression analyses to adjust for confounding factors. Factors with *P* <.10 in the univariate regression analysis were included in the multivariate regression analysis. To avoid multicollinearity, the variance inflation factor was calculated. The Spearman correlation coefficient was calculated to assess the correlation between BC, QDC, QSC, and depression score.

To assess the robustness of our findings to potential unmeasured confounding, we first calculated E-values for the odds ratios (ORs) obtained from the multivariable logistic regression models. The E-value represents the minimum strength of association that an unmeasured confounder would need to have with both the exposure and the outcome, conditional on the measured covariates, to fully explain away the observed association. For ORs <1, the E-value was calculated using the upper limit of the 95% confidence interval (CI). Furthermore, subgroup analyses (by age and gender) and propensity score matching (PSM) were conducted to further verify the robustness of the results. Participants were matched in a 1:1 ratio based on age, gender, ethnicity, health status, only-child status, and single-parent family status. A caliper width of 0.001 was applied to perform caliper and radius matching.

All statistical analyses were performed using R version 4.3.2 (The R Foundation for Statistical Computing, Vienna, Austria; http://www.r-project.org/ (accessed on May 1, 2025)), and *P*-values <.05 were considered significant.

## 4. Results

### 4.1. Descriptive analysis

**Figure 1.**
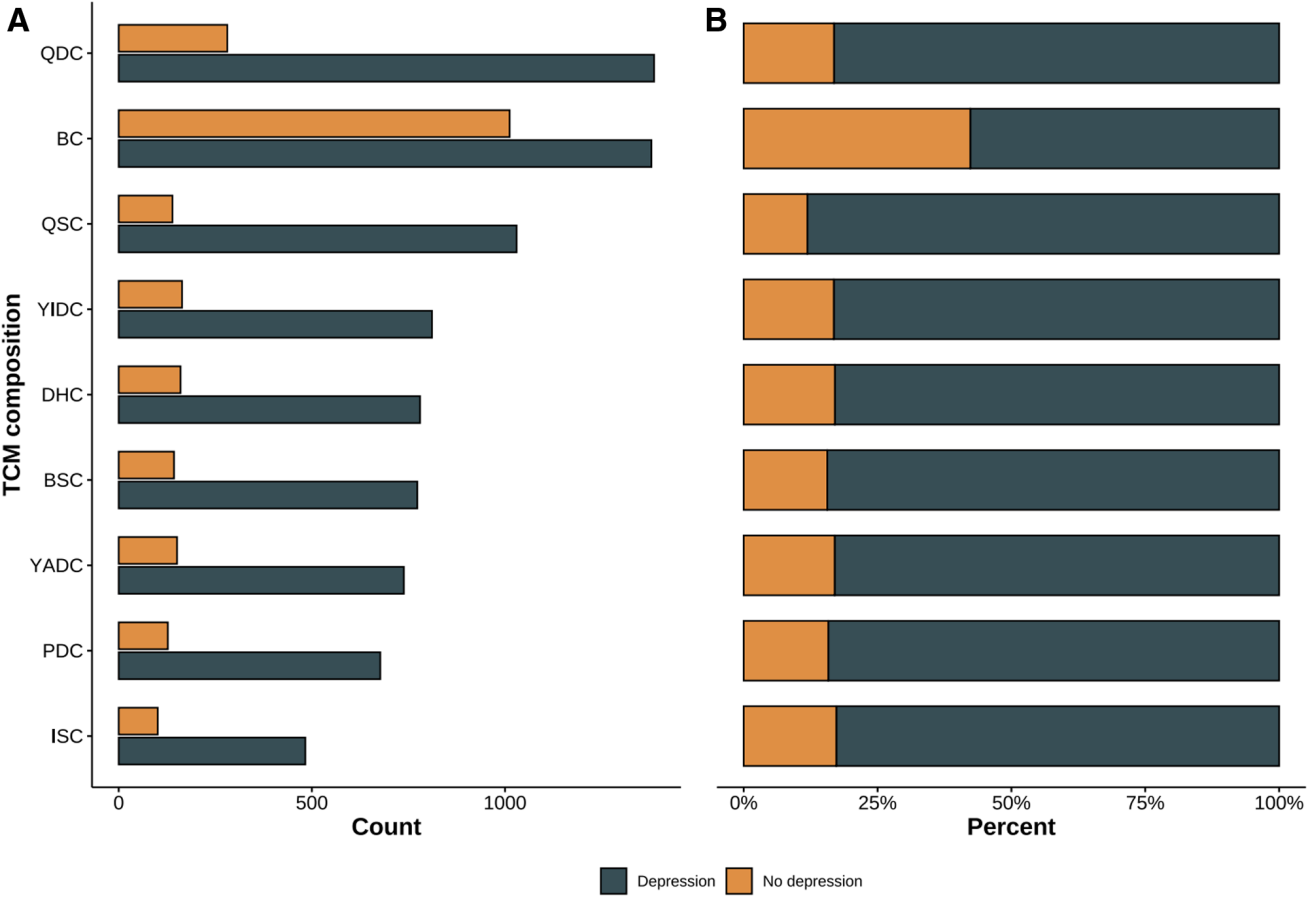
Distribution of traditional Chinese medicine constitutions. BC = balanced constitution, BSC = blood-stasis constitution, DHC = dampness-heat constitution, ISC = inherited special constitution, PDC = phlegm-dampness constitution, QDC = Qi-deficiency constitution, QSC = Qi-stagnation constitution, YADC = Yang-deficiency constitution, YIDC = Yin-deficiency constitution.

A total of 5955 students were included in this study. The mean SDS score was 54.1 ± 11.1, and 3996 students were identified with depression, while 1959 students exhibited no depression (Table 1). The median age of the participants was 18 years (IQR: 17,19), with 3742 male participants (63%) and 2213 female participants (37%). The majority (94%) of students were of Han ethnicity, with a small percentage of students from other ethnic groups. 20% of students came from 1-child families and 724 students (12%) were from single-parent families. 2391 (40%) students are BC, 28% are QDC, 15% are YADC, 16% are YIDC, 14%are PDC, 16% are DHC, 15% are BSC, 20% are QSC and 9.8% are ISC (Fig. 1).

**Table 1:** Characteristics of study participants (N = 5955).

Characteristic	Overall, N = 5955	Depression, N = 3996	No depression, N = 1959	*P*-value*
Age, median (IQR)	18 (17, 19)	18 (17, 19)	18 (17, 19)	<.001
Gender, n (%)				
Female	2213 (37)	1610 (40)	603 (31)	<.001
Male	3742 (63)	2386 (60)	1356 (69)	
Race, n (%)				
Han ethnicity	5599 (94)	3759 (94)	1840 (94)	.826
Ethnic minorities	356 (6.0)	237 (5.9)	119 (6.1)	
From a 1-child family, n (%)	1202 (20)	759 (19)	443 (23)	.001
From a single-parent family, n (%)	724 (12)	518 (13)	206 (11)	.007
Depression score, median (IQR)	56 (46, 63)	61 (55, 64)	43 (38, 46)	<.001
BC score, median (IQR)	66 (53, 75)	63 (50, 72)	72 (63, 81)	<.001
QDC score, median (IQR)	28 (16, 41)	31 (16, 44)	22 (13, 31)	<.001
YADC score, median (IQR)	14 (4, 32)	18 (4, 36)	11 (0, 25)	<.001
YIDC score, median (IQR)	19 (6, 34)	22 (6, 38)	16 (6, 28)	<.001
PDC score, median (IQR)	13 (3, 28)	16 (3, 31)	9 (3, 22)	<.001
DHC score, median (IQR)	17 (4, 33)	17 (4, 33)	13 (0, 25)	<.001
BSC score, median (IQR)	18 (7, 32)	21 (7, 36)	14 (7, 25)	<.001
QSC score, median (IQR)	21 (7, 36)	25 (11, 43)	14 (4, 25)	<.001
ISC score, median (IQR)	14 (4, 29)	18 (7, 29)	14 (4, 25)	<.001
BC, n (%)	2391 (40)	1379 (35)	1012 (52)	<.001
QDC, n (%)	1667 (28)	1386 (35)	281 (14)	<.001
YADC, n (%)	889 (15)	738 (18)	151 (7.7)	<.001
YIDC, n (%)	975 (16)	811 (20)	164 (8.4)	<.001
PDC, n (%)	804 (14)	677 (17)	127 (6.5)	<.001
DHC, n (%)	940 (16)	780 (20)	160 (8.2)	<.001
BSC, n (%)	916 (15)	773 (19)	143 (7.3)	<.001
QSC, n (%)	1169 (20)	1030 (26)	139 (7.1)	<.001
ISC, n (%)	584 (9.8)	483 (12)	101 (5.2)	<.001

BC = balanced constitution, BSC = blood-stasis constitution, DHC = dampness-heat constitution, IQR = interquartile range, ISC = inherited special constitution, PDC = phlegm-dampness constitution, QDC = Qi-deficiency constitution, QSC = Qi-stagnation constitution, YADC = Yang-deficiency constitution, YIDC = Yin-deficiency constitution.

* Wilcoxon rank sum test; Pearson Chi-squared test.

Significant differences were noted between the students with depression and without depression. Participants with depression were more likely to be younger, female, from a single-parent household, and less likely to come from a 1-child family.

In addition, there were significant differences in TCMC between depressed and nondepressed participants (Table 1). Compared to nondepressed participants, depressed participants had a higher proportion of QDC, YADC, YIDC, PDC, DHC, BSC, QSC, ISC, and a lower proportion of BC.

### 4.2. Association between TCMC and depression

Univariate and multivariate logistic regression analyses were conducted with depression as the dependent variable and age, gender, whether only child, whether from a single-parent family, race and the TCMC as independent variables (Table 2). The results of the univariate regression analysis indicated that younger age, male gender, being from a single-child family, and BC (OR = 0.49, 95% CI: 0.44–0.55; *P* <.001) were associated with a lower prevalence of depression. In contrast, Biased constitution types were positively correlated with a higher prevalence of depression. Specifically, QDC (OR = 3.17, 95% CI: 2.75–3.66; *P* <.001), YADC (OR = 2.71, 95% CI: 2.26–3.27; *P* <.001), YIDC (OR = 2.79, 95% CI: 2.34–3.34; *P* <.001), PDC (OR = 2.94, 95% CI: 2.42–3.60; *P* <.001), DHC (OR = 2.73, 95% CI: 2.28–3.27; *P* <.001), BSC (OR = 3.05, 95% CI: 2.53–3.69; *P* <.001), QSC (OR = 4.55, 95% CI: 3.79–5.50; *P* <.001), and ISC (OR = 2.53, 95% CI: 2.04–3.17; *P* <.001) showed significant associations with increased rates of depression.

**Table 2:** Univariate and multivariate Logistic regression analyzes (N = 5955).

Characteristic	Univariable			Multivariawble			
	OR*	95% CI*	*P*-value	OR*	95% CI*	*P*-value	VIF*
Age	0.87	0.84–0.91	**<.001**	0.88	0.84–0.91	**<.001**	1
Gender							
Female	–	–	–	–	–	–	1.1
Male	0.66	0.59–0.74	**<.001**	0.93	0.82–1.05	.262	
From a 1-child family							
No	–	–	–	–	–	–	1.1
Yes	0.8	0.70–0.92	**.001**	0.77	0.67–0.89	**<.001**	
From a single-parent family							
No	–	–	–	–	–	–	1.1
Yes	1.27	1.07–1.51	**.007**	1.27	1.05–1.53	**.012**	
Race							
Ethnic minorities	–	–	–	–	–	–	–
Han ethnicity	1.03	0.82–1.28	.826	–	–	–	–
BC							
No	–	–	–	–	–	–	1.4
Yes	0.49	0.44–0.55	**<.001**	0.83	0.72–0.94	**.004**	
QDC							
No	–	–	–	–	–	–	1.7
Yes	3.17	2.75–3.66	**<.001**	1.68	1.39–2.03	**<.001**	
YADC							
No	–	–	–	–	–	–	1.5
Yes	2.71	2.26–3.27	**<.001**	1.06	0.84–1.35	.601	
YIDC							
No	–	–	–	–	–	–	1.8
Yes	2.79	2.34–3.34	**<.001**	1.03	0.81–1.32	.795	
PDC							
No	–	–	–	–	–	–	1.9
Yes	2.94	2.42–3.60	**<.001**	0.89	0.67–1.19	.444	
DHC							
No	–	–	–	–	–	–	1.7
Yes	2.73	2.28–3.27	**<.001**	1.24	0.98–1.58	.082	
BSC							
No	–	–	–	–	–	–	1.7
Yes	3.05	2.53–3.69	**<.001**	1.13	0.87–1.45	.36	
QSC							
No	–	–	–	–	–	–	1.6
Yes	4.55	3.79–5.50	**<.001**	2.53	2.00–3.23	**<.001**	
ISC							
No	–	–	–	–	–	–	1.4
Yes	2.53	2.04–3.17	**<.001**	0.9	0.69–1.19	.455	

BC = balanced constitution, BSC = blood-stasis constitution, CI = confidence interval, DHC = dampness-heat constitution, ISC = inherited special constitution, OR = odds ratio, PDC = phlegm-dampness constitution, QDC = Qi-deficiency constitution, QSC = Qi-stagnation constitution, VIF = variance inflation factor, YADC = Yang-deficiency constitution, YIDC = Yin-deficiency constitution. Bold values indicate statistical significance (*P* < .05).

**Figure 2.**
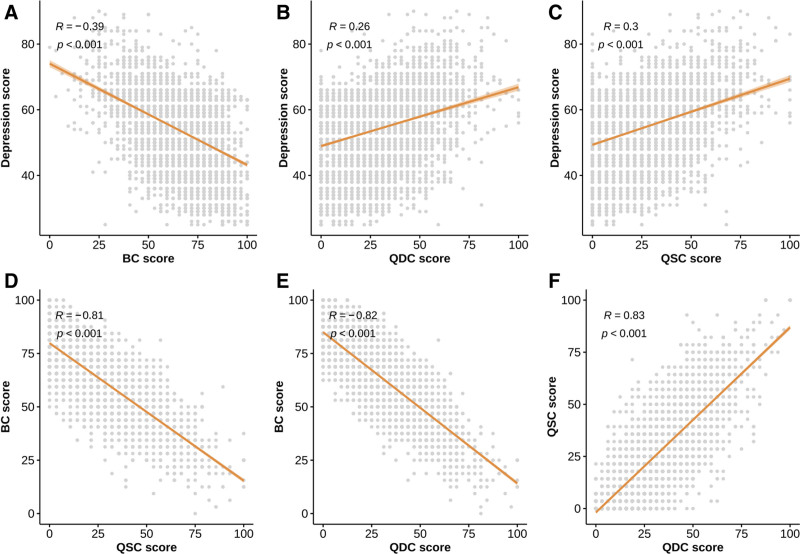
Receiver operating characteristic curves for predicting depression. (A) Model including age and gender; (B) model including age, gender, and TCM Constitutions. TCM = traditional Chinese medicine.

The results of the multivariate regression analysis, accounting for confounding factors, indicated that younger age (OR = 0.88, 95% CI: 0.84–0.91; *P* <.001), being from a single-child family (OR = 0.77, 95% CI: 0.67–0.89; *P* <.001), and BC (OR = 0.83, 95% CI: 0.72–0.94; *P* = .004) were independently linked to a reduced prevalence of depression. Meanwhile, being from a single-parent household (OR = 1.27, 95% CI: 1.05–1.53; *P* = .012), QDC (OR = 1.68, 95% CI: 1.39–2.03; *P* <.001) and QSC (OR = 2.53, 95% CI: 2.00–3.23; *P* <.001) were independently associated with a higher prevalence of depression. To further evaluate the clinical utility of TCMC in predicting depression, we conducted a receiver operating characteristic analysis. The baseline model including only age and gender yielded an area under the curve of 0.576 (95% CI: 0.561–0.591); after adding CCMQ constitution scores to the model, the AUC increased to 0.660 (95% CI: 0.646–0.674) (Fig. 2).

### 4.3. Association between BC, QDC, QSC, and depression

The main concern of this study is the correlation between TCMC and depression. Since multiple TCMC can be present in an individual at the same time, we would like to know the associations between BC, QDC, QSC, and depression. In addition, we wanted to know whether the prevalence of depression was higher in participants with both QDC and QSC than in people with only either QDC or QSC.

**Figure 3.**
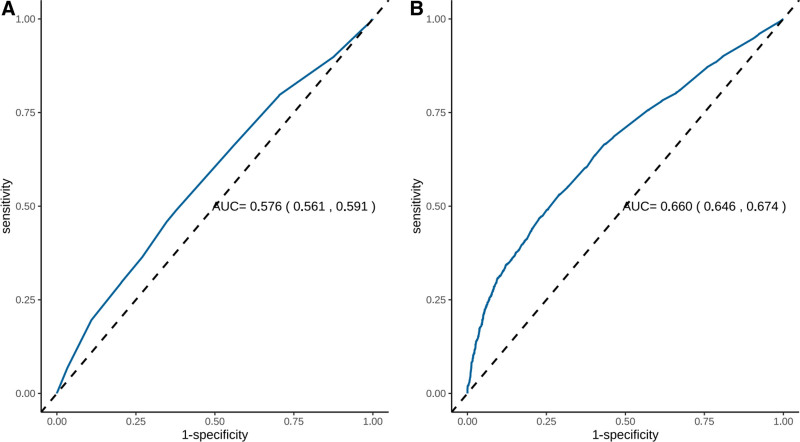
Spearman correlation analysis between depression score, balanced constitution, Qi-deficiency constitution, and Qi-stagnation constitution. BC = balanced constitution, QDC = Qi-deficiency constitution, QSC = Qi-stagnation constitution.

As a result, we conducted Spearman correlation analysis to examine the relationship between depression score and score on BC, QDC, and QSC. Significant correlation was determined between depression score and constitution score (all *P* <.001). Specifically, depression score was negatively correlated with BC (*r*_*s*_ = −0.39) and positively correlated with QSC (*r*_*s*_ = 0.30) and QDC (*r*_*s*_ = 0.26). Furthermore, we also found that QDC and QSC exhibited a strong positive correlation (*r*_*s*_ = 0.83), while both QDC (*r*_*s*_ = −0.82) and QSC (*r*_*s*_ = −0.81) showed a strong negative correlation with BC (Fig. 3).

**Figure 4.**
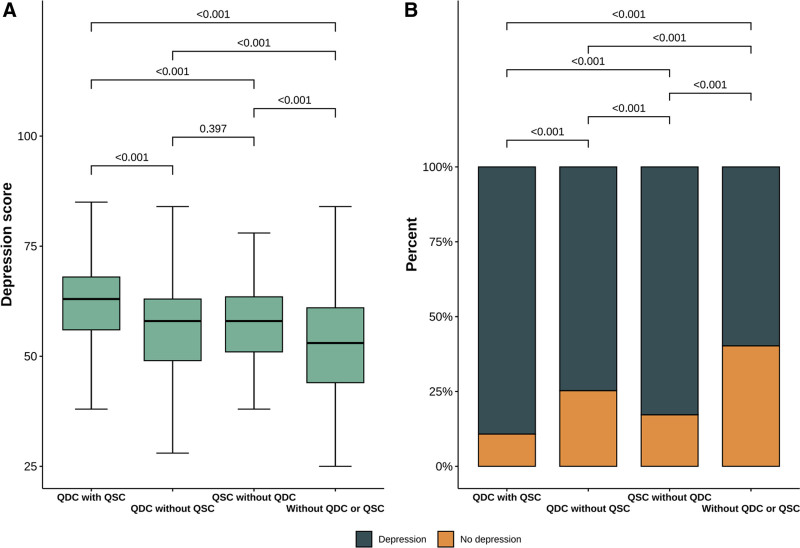
Post hoc analysis for groups divided by Qi-deficiency constitution and Qi-stagnation constitution. QDC = Qi-deficiency constitution, QSC = Qi-stagnation constitution.

As shown in Figure 4, participants with QDC and QSC had significantly higher depression scores than the other 3 groups (All *P* <.001). Participants with either only QDC or QSC had significantly higher depression scores than participants without QDC and QSC (All *P* <.001). In addition, to better capture the overlapping nature of constitutions commonly observed in TCM clinical practice, we performed a sensitivity analysis by categorizing participants into 4 groups: without QDC or QSC, QDC only, QSC only, and both QDC and QSC. As shown in Table 3, participants with both QDC and QSC exhibited the highest proportions of moderate (47%) and severe (18%) depression. Furthermore, as presented in Table 4, the multivariate regression model revealed that, compared with participants without QDC or QSC, those with QDC only, QSC only, or both QDC and QSC had a significantly higher prevalence of depression.

**Table 3:** The relationship between qi-deficiency constitution, qi-stagnation constitution and depression (N = 5955).

Characteristic	QDC with QSC, N = 966	QDC without QSC, N = 701	QSC without QDC, N = 203	Without QDC and QSC, N = 4085	*P*-value*
Depression, n (%)	862 (89)	524 (75)	168 (83)	2442 (60)	**<.001**
Depression score, median (IQR)	63 (56, 68)	58 (49, 63)	58 (51, 64)	53 (44, 61)	**<.001**
Degree of depression, n (%)					
No depression	104 (11)	177 (25)	35 (17)	1643 (40)	**<.001**
Mild depression	235 (24)	239 (34)	81 (40)	1158 (28)	
Moderate depression	451 (47)	242 (35)	72 (35)	1227 (30)	
Severe depression	176 (18)	43 (6.1)	15 (7.4)	57 (1.4)	

IQR = interquartile range, QDC = Qi-deficiency constitution, QSC = Qi-stagnation constitution.

* Pearson Chi-squared test; Kruskal–Wallis rank sum test. Bold values indicate statistical significance (*P* < .05).

**Table 4:** Regression analysis of the relationship between qi-deficiency constitution, qi-stagnation constitution and depression (N = 5955).

Characteristic	Univariable			Multivariable*		
	OR	95% CI	*P*-value	OR	95% CI	*P*-value
QDC/QSC category						
Without QDC or QSC	–	–	–	–	–	–
QDC without QSC	1.99	1.66–2.39	**<.001**	1.7	1.39–2.09	**<.001**
QSC without QDC	3.23	2.26–4.74	**<.001**	2.66	1.84–3.96	**<.001**
QDC with QSC	5.58	4.53–6.93	**<.001**	4.19	3.17–5.61	**<.001**

CI = confidence interval, OR = odds ratio, QDC = Qi-deficiency constitution, QSC = Qi-stagnation constitution. Bold values indicate statistical significance (*P* < .05).

* Adjusted for age, gender, whether from a 1-child family, whether from a single-parent family, race, and other traditional Chinese medicine constitutions.

## 5. Sensitivity analysis

To further assess the robustness of the observed associations to potential unmeasured confounding, E-values were calculated based on the ORs from the multivariable logistic regression models, which included all covariates with *P*-values <0.1 in the univariate analyses. For BC, the E-value was 1.43 (based on the upper limit of the 95% CI: 1.21); for QSC, the E-value was 2.56 (95% CI lower limit: 2.18); and for QDC, the E-value was 1.92 (95% CI lower limit: 1.64). These results suggest that an unmeasured confounder would need to be associated with both the exposure and outcome by a risk ratio of at least this magnitude to fully explain away the observed associations, indicating moderate robustness (Table S1, Supplemental Digital Content, https://links.lww.com/MD/R189).

To enhance the robustness of the results, the PSM method was used, which resulted in balanced groups with underlying characteristics. PSM resulted in 3872 matched individuals. Comparison of the balance of each factor between the depressed and nondepressed groups can be judged by the standardized mean difference (SMD). SMD >0.1 is regarded as an unbalanced distribution. Figure S1, Supplemental Digital Content, https://links.lww.com/MD/R189 represents the change of SMD before and after PSM, which shows that the distribution of age and gender is not balanced before matching. The SMD after matching of these factors were 0 and the distribution is balanced. Figure S2, Supplemental Digital Content, https://links.lww.com/MD/R189 illustrates the density distribution of propensity score values in the depressed and nondepressed groups before and after matching. There was a large area of overlap between the distributions of the depressed and nondepressed groups before matching. The propensity scores of the 2 groups were much closer after matching. After matching, the conditional multivariate regression results showed that there were significant associations between 2 constitution types (QDC: OR = 1.20, 95% CI: 1.05–1.38, *P* = .009;QSC: OR = 1.37, 95% CI: 1.18–1.60, *P* <.001) and depression, which was similar to the results before PSM analysis (Table S2, Supplemental Digital Content, https://links.lww.com/MD/R189). Although the relationship between BC and depression was not significant, there was a negative correlation trend observed (BC: OR = 0.90, 95% CI: 0.81–1.01, *P* = .065) (Table S2, Supplemental Digital Content, https://links.lww.com/MD/R189).

We further performed subgroup analyses (Table S3, Supplemental Digital Content, https://links.lww.com/MD/R189). The results showed that the association between QSC and depression was stronger among participants aged ≤18 years, with an OR of 3.31 (95% CI: 2.18–5.02) (*P* for interaction = .026). However, the association between QSC and depression remained statistically significant across all age strata. In addition, a similar trend was observed for QDC (*P* for interaction = .055), suggesting that the influence of TCMC on depressive symptoms may be more pronounced among adolescents or younger individuals. In addition, in the age-based sensitivity analysis, the results of the multivariate regression among students aged 14 to 19 years (n = 5081) were consistent with those of the main analysis (BC: OR = 0.84, 95% CI: 0.73–0.94, *P* = .016; QDC: OR = 1.58, 95% CI: 1.29–1.95, *P* <.001; QSC: OR = 2.69, 95% CI: 2.08–3.49, *P* <.001) (Table S4, Supplemental Digital Content, https://links.lww.com/MD/R189). Furthermore, we conducted an additional analysis including the 39 students who had been previously excluded for being over the age of 24. The results remained consistent with the primary analysis, supporting the robustness of the findings (Multivariable model: BC: OR = 0.83, 95% CI: 0.72–0.94, *P* = .004; QDC: OR = 1.69, 95% CI: 1.40–2.04, *P* <.001; QSC: OR = 2.53, 95% CI: 2.00–3.21, *P* <.001) (Table S5, Supplemental Digital Content, https://links.lww.com/MD/R189). We further conducted a sensitivity analysis using a higher cutoff (SDS ≥60) to define clinically significant depression. The results showed that both QSC and QDC remained significantly associated with clinically significant depression (QSC: OR = 2.05, 95% CI: 1.71–2.47; QDC: OR = 1.60, 95% CI: 1.35–1.89; all *P* <.001). These findings indicate that the associations between TCMC and depressive symptoms are not limited to subclinical distress, but also extend to more severe, clinically meaningful depressive conditions (Table S6, Supplemental Digital Content, https://links.lww.com/MD/R189).

## 6. Discussion

Our study explored the relationship between depression and TCMC in adolescents. The results showed that BC was associated with a lower prevalence of depression, while QDC and QSC were associated with a higher prevalence of depression in adolescent population.

TCMC is grounded in the concept that each individual possesses a unique, relatively stable constitution – an integrated profile of physical structure, physiological function, and psychological tendencies – that both shapes and is shaped by lifestyle and environmental factors.^[38]^ Over centuries of clinical observation, TCM practitioners have noted that deviations from a “balanced” constitution predispose individuals to specific patterns of vulnerability: for example, Qi-Deficiency–type individuals often experience chronic fatigue and poor immunity,^[39,40]^ while those with Phlegm-Dampness–type may have metabolic disturbances and a tendency toward obesity.^[41]^ Once a constitution type is determined, a suite of TCM interventions can be tailored accordingly. For instance, Xiao Yao San and related herbal formulas are prescribed to “soothe Liver-Qi stagnation” and alleviate emotional constraint in QSCs.^[42]^

This study provides evidence for the application of the TCMC theory in addressing adolescent depression. Yap et al meta-analysis,^[43]^ which included 13 studies, revealed that the pooled ORs for developing depression with Qi-stagnation, Qi-deficiency, Yang-deficiency, Yin-deficiency, and Balanced constitutions were as follows: 3.12 (95% CI: 1.80–5.40; I2 = 94%), 2.15 (95% CI: 1.54–3.01; I2 = 89%), 1.89 (95% CI: 0.71–5.03; I2 = 81%), 1.41 (95% CI: 0.91–2.20; I2 = 57%), and 0.60 (95% CI: 0.40–0.90; I2 = 94%), respectively. These findings are consistent with our results, indicating that BC is negatively associated with depression, while biased constitutions are positively correlated with depressive symptoms. However, there remains some controversy regarding which specific biased constitutions are linked to depression. A study conducted at Turpan Vocational Technical College involving 532 vocational college students found that QSCs, QDCs, and YADCs were associated with more severe depressive symptoms.^[20]^ Another study involving 5311 freshmen from a vocational college in Shenzhen found that BC (B = −1.65, *P* <.01) served as a protective factor against depression, while Qi-deficiency (B = 0.47, *P* <.05), Blood-stasis (B = 0.55, *P* <.05), and Qi-stagnation (B = 1.43, *P* <.01) constitutions were identified as risk factors for depressive symptoms.^[19]^ The discrepancies across these studies may be attributable to differences in study design, such as variations in study populations and measurement tools.

In TCM, Qi is a substance that runs continuously in the body and is equivalent to energy or signal in modern medicine.^[44]^ People with QDC are characterized by weakness, fatigue, a reduced voice frequency, shortness of breath, reticence, introverted traits, and timidity, similar to depressive symptoms. Individuals with Qi-deficiency exhibit weak cognitive control abilities and a low vagal tone.^[45,46]^ People with Qi-deficiency tend to have a reduced diversity of gut microbiota and show distinct microbial profiles in comparison to individuals with BC.^[40]^ In the Qi-deficiency rat model, various changes in biomarkers have been observed, mainly involving alterations in energy metabolism, amino acid metabolism, tryptophan metabolism, purine metabolism and pyrimidine metabolism.^[47]^ A network pharmacology study has revealed that Qi-deficiency involves alterations in biological functions such as immune regulation, oxidative stress, and lipid metabolism.^[48]^ In thousands of years of medical practice, TCM has discovered a series of Qi-tonifying herbal plants that can be used to treat Qi-deficiency. Many modern studies suggest that these herbal medicines have immunomodulatory effects,^[49]^ enhance mitochondrial energy metabolism, provide antioxidant properties,^[50]^ and regulate gut microbiota metabolism.^[51]^ This may explain why many Qi-tonifying herbs have antidepressant effects.^[52]^

The QSC is associated with a higher prevalence of depression among adolescents. This constitution is characterized by a prolonged state of sluggish energy metabolism or signal transduction within the body.^[53]^ The Qi-stagnation is a pattern characterized by a sensation of obstruction in the throat, a sensation of ear tube obstruction, abdominal distension due to intestinal gas retention, depressive state, or intractable pain. It may be explained by the hindered Qi movement of related organs.^[54]^ People with this constitution are more prone to stress and are more likely to experience emotional distress when they encounter negative life events.^[9]^ In TCM, the QSC is characterized by impaired Qi flow and emotional constraint, manifesting as emotional stasis and prolonged melancholy. In Western cognitive psychology, “rumination” and “negative thinking patterns” describe individuals repeatedly falling into cycles of negative thoughts and emotions,^[55]^ unable to break free from a pessimistic thought spiral – concepts that closely mirror those of QSC.^[44]^ People with a high tendency toward rumination or frequent negative automatic thoughts are more likely to develop depression after adverse life events.^[56,57]^ Therefore, the elevated depression prevalence observed in individuals with QSC may be linked to their propensity for rumination and negative thinking patterns. Metabolomics research has found a connection between Qi stagnation with abnormal branched-chain amino acids, and energy metabolism.^[58]^ MDD patients with Qi-stagnation may represent a subtype of MDD characterized by abnormalities in the biosynthesis of monoamine and amino acid neurotransmitters, closely associated with stress-related pathophysiology.^[58]^ Herbal medicine for Qi-stagnation are effective for depression treatment and have promising applications. Yueju is a well-known traditional Chinese herbal formula commonly used to treat Qi-stagnation.^[59]^ Studies conducted on animals have suggested that Yueju has the potential to treat depression by reversing the down-regulation of depression-related proteins PKA and CREB in mice.^[60,61]^

**Figure 5.**
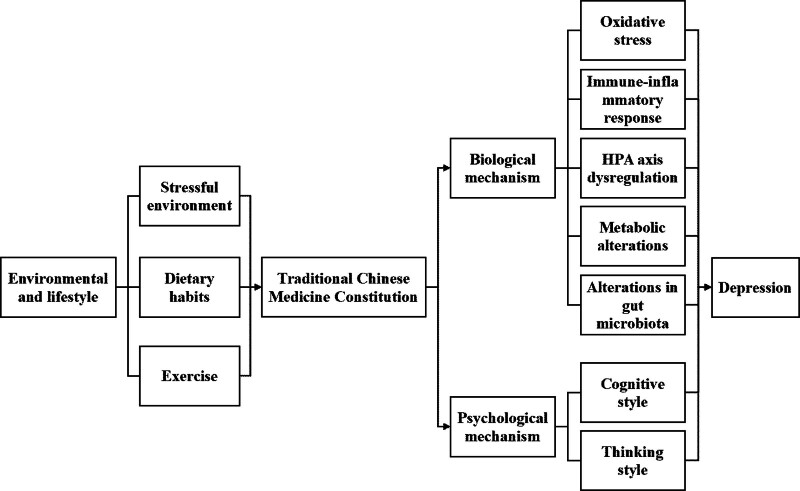
Conceptual model linking TCM Constitution types with environmental factors, lifestyle, and biopsychosocial mechanisms underlying depression. TCM = = traditional Chinese medicine.

The term BC refers to a state of physical well-being that is characterized by moderate physical appearance, a rosy complexion, and high energy levels.^[62]^ According to our research, adolescents with a BC have a low prevalence of depression. Individuals with a BC are better equipped to adapt to their environment, thereby making them less vulnerable to the impact of negative events and environmental disturbances. This study suggests that in adolescent health management, BC can be considered as a target to strive for. To facilitate readers’ understanding, we developed a conceptual figure (Fig. 5) to illustrate how different TCMC types may be interconnected with environmental factors, lifestyle, and multiple biopsychosocial mechanisms.

Although adolescent depression has become a major global public health concern and numerous studies have focused on its early identification, diagnostic criteria, and treatment strategies, many knowledge gaps remain in this field. Due to the shortage of mental health professionals and the often hidden nature of depressive symptoms in adolescents, timely recognition and intervention continue to be major challenges.^[63]^ Some experts have suggested that, given the complexity and high cost of treating adolescent depression, greater emphasis should be placed on preventive strategies targeting high-risk populations.^[64]^ Against this background, the CCMQ serves as a simple, cost-effective, and widely applicable assessment tool that can help identify adolescents with biased or imbalanced constitution types. Such constitutions often reflect underlying vulnerabilities in emotional regulation and physiological balance. Early identification of these constitutional characteristics provides an opportunity for health education, lifestyle modification, and preventive TCM-based interventions, which may help reduce the risk of depression and other stress-related disorders.

This study has a couple of limitations. First, our participants were students from a single vocational school in Zhuhai, China, which limits generalizability. Vocational students may differ from peers in other educational settings regarding socioeconomic status, psychological stress, and cultural backgrounds, potentially affecting TCMC distribution and depression. Future multi-center studies involving more diverse adolescent groups across different geographic and socioeconomic backgrounds would help validate and expand on these findings. Secondly, due to its cross-sectional design, this study cannot determine a causal relationship between TCMC and depression. Although significant associations were observed, the directionality of these relationships remains uncertain. For example, depressive symptoms may influence how individuals perceive or report their constitutional traits. Future longitudinal studies are warranted to track changes in TCMC types and depressive symptoms over time, which would provide stronger evidence for establishing causal relationships, and further assess the clinical value of constitution-based screening and interventions. Moreover, the use of self-administered electronic questionnaires is convenient, there could be limitations in terms of accuracy, such as social desirability bias or incomplete self-assessment. Although the CCMQ is a widely used and reliable assessment tool, it cannot capture the individualized pattern differentiation of TCMC in the same way that professional TCM physicians can through traditional diagnostic methods. Therefore, future research should incorporate professional evaluations by TCM practitioners, including tongue and pulse diagnostics, to achieve more precise constitution classification. Additionally, this study did not measure cultural influences, and there may be unmeasured confounding factors that were not accounted for in the analysis.

In conclusion, BC were associated with a lower prevalence of depression, while QDC and QSC were independently associated with a higher prevalence of depression. Further research is needed to better understand whether these constitutional factors may contribute to or influence the development of depression during adolescence. Integrating CCMQ into clinical assessments may provide valuable insights for identifying at-risk individuals and guiding personalized treatments for depression in adolescents.

## Author contributions

**Conceptualization:** Jun Chen.

**Data curation:** Xingwei Fang.

**Formal analysis:** Xingwei Fang.

**Funding acquisition:** Jun Chen.

**Investigation:** Jun Chen.

**Methodology:** Jun Chen.

**Project administration:** Min Lu, Jun Chen.

**Resources:** Jun Chen.

**Supervision:** Bin Luo, Min Lu, Zhangjin Zhang, Jun Chen.

**Validation:** Jun Chen.

**Visualization:** Jiahui Yin, Xingwei Fang.

**Writing – original draft:** Yizhao Hao, Zhe Huang.

**Writing – review & editing:** Jiahui Yin, Yueqi Zhang, Yuanyuan Liu, Jingli Zhang, Xingwei Fang, Zhongli Lan, Bin Luo, Min Lu, Zhangjin Zhang, Jun Chen.

## Supplementary Material



## References

[1] Patton GC Sawyer SM Santelli JS. Our future: a lancet commission on adolescent health and wellbeing. Lancet. 2016;387:2423–78. 27174304 10.1016/S0140-6736(16)00579-1 PMC5832967

[2] Miller L Campo JV. Depression in adolescents. N Engl J Med. 2021;385:445–9. 34320289 10.1056/NEJMra2033475

[3] Gunlicks-Stoessel M Klimes-Dougan B VanZomeren A Ma S. Developing a data-driven algorithm for guiding selection between cognitive behavioral therapy, fluoxetine, and combination treatment for adolescent depression. Transl Psychiatry. 2020;10:321. 32958758 10.1038/s41398-020-01005-y PMC7506003

[4] Chaplin AB Daniels NF Ples D. Longitudinal association between cardiovascular risk factors and depression in young people: a systematic review and meta-analysis of cohort studies. Psychol Med. 2023;53:1049–59. 34167604 10.1017/S0033291721002488 PMC9975997

[5] Kwong ASF Morris TT Pearson RM. Polygenic risk for depression, anxiety and neuroticism are associated with the severity and rate of change in depressive symptoms across adolescence. J Child Psychol Psychiatry. 2021;62:1462–74. 33778956 10.1111/jcpp.13422

[6] Wahid SS Ottman K Hudhud R. Identifying risk factors and detection strategies for adolescent depression in diverse global settings: a delphi consensus study. J Affect Disord. 2021;279:66–74. 33039776 10.1016/j.jad.2020.09.098 PMC7758738

[7] Liu ZL Liu JP Zhang AL. Chinese herbal medicines for hypercholesterolemia. Cochrane Database Syst Rev. 2011;2011:CD008305. 21735427 10.1002/14651858.CD008305.pub2 PMC3402023

[8] Wang Q. Traditional Chinese Medicine Body Consititution. Beijing: People’s Medical Publishing House; 2009.

[9] Wang Q. Classification and diagnosis basis of nine basic constitutions in Chinese medicine. J Beijing Univ Tradit Chin Med. 2005;1–8.

[10] Hong SW Yoo J-W Bose S. Understanding the molecular aspects of oriental obesity pattern differentiation using DNA microarray. J Transl Med. 2015;13:331. 26482123 10.1186/s12967-015-0692-9 PMC4617455

[11] Liang X Wang Q Jiang Z. Clinical research linking traditional Chinese medicine constitution types with diseases: a literature review of 1639 observational studies. J Tradit Chin Med. 2020;40:690–702. 32744037 10.19852/j.cnki.jtcm.2020.04.019

[12] Yaqi W Weibo Z Yixing W. Traditional Chinese medicine constitution among patients with allergic rhinitis and its correlation with anxiety and depression. J Tradit Chin Med. 2023;43:1252–8. 37946488 10.19852/j.cnki.jtcm.20230919.001 PMC10623246

[13] Sun Z Ping P Li Y. Relationships between traditional Chinese medicine constitution and age-related cognitive decline in Chinese centenarians. Front Aging Neurosci. 2022;14:870442. 35615593 10.3389/fnagi.2022.870442 PMC9126494

[14] Zhang T Luo H Wei D. Traditional Chinese medicine constitution correlated with ischemic stroke: a systematic review and meta-analysis. Evid Based Complement Alternat Med. 2021;2021:5524925. 34257683 10.1155/2021/5524925 PMC8253626

[15] Ma Y-L Yao H Yang W-J Ren X-X Teng L Yang M-C. Correlation between traditional Chinese medicine constitution and dyslipidemia: a systematic review and meta-analysis. Evid Based Complement Alternat Med. 2017;2017:1896746. 29234371 10.1155/2017/1896746 PMC5671713

[16] Bai F Luo H Wang L. A Meta-analysis of the association between diabetes mellitus and traditional Chinese medicine constitution. Evid Based Complement Alternat Med. 2021;2021:6390530. 34394389 10.1155/2021/6390530 PMC8357480

[17] Zhu K Guo Y Zhao C. Etiology exploration of non-alcoholic fatty liver disease from traditional Chinese medicine constitution perspective: a cross-sectional study. Front Public Health. 2021;9:635818. 34055713 10.3389/fpubh.2021.635818 PMC8149586

[18] Cao J Chen P Dai Z. Study on the correlation between the depression of students in Huang lakes university town wuhan and the constitution of Chinese medicine. Chin J Ethnomed Ethnopharm. 2021;30:16–9.

[19] Zhang G Li F Zhang J Wu H. Analysis of the physique and depression of 5 311 freshmen in higher vocational colleges. Chin J Tradit Chin Med Pharm. 2022;37:7409–12.

[20] Yu X Gao J. Correlation analysis between depressive symptoms and traditional Chinese medicine constitution of higher vocational college students. Pol Sci Consult. 2024;9:60–3.

[21] Thapar A Eyre O Patel V Brent D. Depression in young people. Lancet (London, England). 2022;400:617–31. 35940184 10.1016/S0140-6736(22)01012-1

[22] Lu T Yan J Chang J. Valid and convenient questionnaire assessment of chinese body constitution: item characteristics, reliability, and construct validation. Patient Prefer Adherence. 2022;16:1875–84. 35942226 10.2147/PPA.S373512 PMC9356699

[23] Zhu Y Shi H Yu X. Comparison of the performance of three versions of constitution of chinese medicine questionnaire for healthy populations. Chin General Pract. 2019;22:4381–7.

[24] Zhu Y Wang Q Xue H Orlkasa O. Preliminary assessement on perfornmnce of constitution in Chinese medicine questionnaire. Chin J Clin Rehabil. 2006;10:15–7.

[25] China Association of Chinese Medicine. Classification and determination of TCM constitution (ZYYXH/T157-2009). World J Int Trad Western Med. 2009;4:303–4.

[26] Wang C Zhang H Nie X. Traditional Chinese medicine constitution and sarcopenia: a cross-sectional study. Front Public Health. 2024;12:1368933. 39114511 10.3389/fpubh.2024.1368933 PMC11304350

[27] Sun W Bai M Wang J. Machine learning-assisted rapid determination for traditional Chinese medicine constitution. Chin Med. 2024;19:127. 39278905 10.1186/s13020-024-00992-0 PMC11403957

[28] Wang J Zhang C Zhang Y. Protocol for a nested case-control study: identifying neuroimaging biomarkers for the progression of subclinical depression and qi-stagnation constitution to major depressive disorder in adolescents. Front Psychiatry. 2024;15:1516846. 39906680 10.3389/fpsyt.2024.1516846 PMC11790624

[29] Gong J He Y Wang S Liu J. Emotion regulation and depressive symptoms mediate the association between schizotypal personality traits and suicidality in Chinese college students. Arch Suicide Res. 2022;26:614–25. 32924826 10.1080/13811118.2020.1818655

[30] Nie J Zhang W Liu Y. Exploring depression, self-esteem and verbal fluency with different degrees of internet addiction among Chinese college students. Compr Psychiatry. 2017;72:114–20. 27810547 10.1016/j.comppsych.2016.10.006

[31] Lee HC Chiu HFK Wing YK Leung CM Kwong PK Chung DWS. The Zung self-rating depression scale: screening for depression among the Hong Kong Chinese elderly. J Geriatr Psychiatry Neurol. 1994;7:216–20. 7826489 10.1177/089198879400700404

[32] Leung KK Lue BH Lee MB Tang LY. Screening of depression in patients with chronic medical diseases in a primary care setting. Fam Pract. 1998;15:67–75. 9527300 10.1093/fampra/15.1.67

[33] Hu WM Yin XY Yin XL. Prevalence, social-demographic and cognitive correlates of depression in Chinese psychiatric medical staff. J Affect Disord. 2020;263:60–3. 31818797 10.1016/j.jad.2019.11.133

[34] Liao J Zhu S Li X. Anxiety and depression in paradoxical insomnia: a case–control study. Neuropsychiatr Dis Treat. 2018;14:231–8. 29386896 10.2147/NDT.S156058 PMC5764296

[35] Shao R He P Ling B. Prevalence of depression and anxiety and correlations between depression, anxiety, family functioning, social support and coping styles among Chinese medical students. BMC Psychol. 2020;8:38. 32321593 10.1186/s40359-020-00402-8 PMC7178943

[36] Dunstan DA Scott N. Clarification of the cut-off score for Zung’s self-rating depression scale. BMC Psychiatry. 2019;19:177. 31185948 10.1186/s12888-019-2161-0 PMC6558728

[37] Suzuki T Shiga T Kuwahara K. Depression and outcomes in hospitalized Japanese patients with cardiovascular disease – prospective single-center observational study -: – prospective single-center observational study –. circ j. 2011;75:2465–73. 21791870 10.1253/circj.cj-11-0140

[38] Sun Y Zhao Y Xue SA Chen J. The theory development of traditional Chinese medicine constitution: a review. J Trad Chin Med Sci. 2018;5:16–28.

[39] Kim J Ku B Kim KH. Validation of the qi blood yin yang deficiency questionnaire on chronic fatigue. Chin Med. 2016;11:24. 27141228 10.1186/s13020-016-0092-y PMC4852426

[40] Ma K Chen J Kuang L. Qi-deficiency related increases in disease susceptibility are potentially mediated by the intestinal microbiota. Evid Based Complement Alternat Med. 2018;2018:1304397. 30425748 10.1155/2018/1304397 PMC6218746

[41] Wang J Wang Q Li L. Phlegm-dampness constitution: genomics, susceptibility, adjustment and treatment with traditional Chinese medicine. Am J Chin Med. 2013;41:253–62. 23548117 10.1142/S0192415X13500183

[42] Wang Y-T Wang X-L Wang Z-Z Lei L Hu D Zhang Y. Antidepressant effects of the traditional Chinese herbal formula Xiao-Yao-San and its bioactive ingredients. Phytomedicine. 2023;109:154558. 36610123 10.1016/j.phymed.2022.154558

[43] Yap SY Ng FL Subramaniam M Lim YM Foo CN. Traditional Chinese medicine body constitutions as predictors for depression: a systematic review and meta-analysis. Behav Sci (Basel, Switzerland). 2022;12:423. 10.3390/bs12110423 PMC9687208 36354400

[44] Huang H Song Q Chen J. The role of Qi-Stagnation constitution and emotion regulation in the association between childhood maltreatment and depression in Chinese college students. Front Psychiatry. 2022;13:825198. 35599766 10.3389/fpsyt.2022.825198 PMC9114459

[45] Lin H-Y Zhao Y-P Xu G-P. Weaker cognitive control abilities of Pi (Spleen) qi-deficient individuals supported Chinese medicine diagnosis. Chin J Integr Med. 2017. 10.1007/s11655-017-2967-x 28755078

[46] Olivera-Toro A Fossion R Li L. Changes in heart rate variability in patients with spleen-qi deficiency syndrome. J Acupunct Meridian Stud. 2019;12:111–21. 31351997 10.1016/j.jams.2019.07.002

[47] Liu W-P Li C-Y Huang J. [Identification of biomarkers in urine of rats with spleen Qi deficiency and biological significance]. Zhongguo Zhong Yao Za Zhi. 2017;42:4855–63. 29493158 10.19540/j.cnki.cjcmm.20170919.003

[48] Wang X Wu M Lai X. Network pharmacology to uncover the biological basis of spleen qi deficiency syndrome and herbal treatment. Oxid Med Cell Longev. 2020;2020:2974268. 32908629 10.1155/2020/2974268 PMC7474375

[49] Huang L Ye M Wu J Liu W Chen H Rui W. A metabonomics and lipidomics based network pharmacology study of qi-tonifying effects of honey-processed Astragalus on spleen qi deficiency rats. J Chromatogr B Analyt Technol Biomed Life Sci. 2020;1146:122102. 10.1016/j.jchromb.2020.122102 32330807

[50] Tian J Huang Y Wu T. The use of Chinese Yang/Qi-Invigorating tonic botanical drugs/herbal formulations in ameliorating chronic kidney disease by enhancing mitochondrial function. Front Pharmacol. 2021;12:622498. 34248614 10.3389/fphar.2021.622498 PMC8264145

[51] Wang N Huang X Li T. Application of RRLC-QTOF-MS-based metabonomics and UPE for investigating Spleen-Qi deficiency syndrome with Panax ginseng treatment. J Ethnopharmacol. 2020;256:112822. 32247146 10.1016/j.jep.2020.112822

[52] Zhao Y Teng J Yang H. [Analysis on medication regularity of modern traditional Chinese medicines in treating melancholia based on data mining technology]. Zhongguo Zhong Yao Za Zhi. 2015;40:2042–6. 26390670

[53] Okitsu R Iwasaki K Monma Y. Development of a questionnaire for the diagnosis of Qi stagnation. Complement Ther Med. 2012;20:207–17. 22579432 10.1016/j.ctim.2011.12.005

[54] Maeda-Minami A Ihara K Yoshino T Horiba Y Mimura M Watanabe K. A prediction model of qi stagnation: a prospective observational study referring to two existing models. Comput Biol Med. 2022;146:105619. 35598353 10.1016/j.compbiomed.2022.105619

[55] Ehring T. Thinking too much: rumination and psychopathology. World Psychiatr. 2021;20:441–2. 10.1002/wps.20910 PMC8429319 34505392

[56] Zhou H-X Chen X Shen Y-Q. Rumination and the default mode network: meta-analysis of brain imaging studies and implications for depression. Neuroimage. 2020;206:116287. 31655111 10.1016/j.neuroimage.2019.116287

[57] Spinhoven P van Hemert AM Penninx BW. Repetitive negative thinking as a predictor of depression and anxiety: a longitudinal cohort study. J Affect Disord. 2018;241:216–25. 30138805 10.1016/j.jad.2018.08.037

[58] Liu L-Y Zhang H-J Luo L-Y. Blood and urinary metabolomic evidence validating traditional Chinese medicine diagnostic classification of major depressive disorder. Chin Med. 2018;13:53. 30386416 10.1186/s13020-018-0211-z PMC6203264

[59] Zhang W Tang S-H Zhang Y. [Study on Yueju Wan for “different diseases with same treatment” based on integrative pharmacology]. Zhongguo Zhong Yao Za Zhi. 2018;43:1352–9. 29728023 10.19540/j.cnki.cjcmm.20180123.001

[60] Xue W Wang W Gong T. PKA-CREB-BDNF signaling regulated long lasting antidepressant activities of Yueju but not ketamine. Sci Rep. 2016;6:26331. 27197752 10.1038/srep26331 PMC4873804

[61] Zou Z Chen Y Shen Q Guo X Zhang Y Chen G. Neural plasticity associated with hippocampal PKA-CREB and NMDA signaling is involved in the antidepressant effect of repeated low dose of Yueju Pill on chronic mouse model of learned helplessness. Neural Plast. 2017;2017:9160515. 29075536 10.1155/2017/9160515 PMC5623799

[62] Zhu H Qin S Wu M. Association between weekend catch-up sleep and cardiovascular disease: evidence from the national health and nutrition examination surveys 2017-2018. Sleep Health. 2024;10:98–103. 38000943 10.1016/j.sleh.2023.09.006

[63] Reese JM Hofmann S Barone MA Serwint J. Training residents in adolescent depression. Med Educ. 2021;55:1328–9. 34462949 10.1111/medu.14650

[64] Muñoz RF Cuijpers P Smit F Barrera AZ Leykin Y. Prevention of major depression. Annu Rev Clin Psychol. 2010;6:181–212. 20192789 10.1146/annurev-clinpsy-033109-132040

