# Supplementary material for: Association between traditional Chinese medicine constitution and depression in adolescents: A cross-sectional study
Source: Medicine (Baltimore). 2026 Jan 23;105(4):e47310. doi: 10.1097/MD.0000000000047310 (PMC12851703; doi:10.1097/MD.0000000000047310)
Supplement: Supplementary file 1 [file medi-105-e47310-s001.docx]

**Table S1: E-value^1^ for the association between Traditional Chinese Medicine Constitution and depression.**

| **Characteristic** | **OR** | **95% CI** | **E-value for OR** | **E-value for CI** |
| --- | --- | --- | --- | --- |
| **BC** | 0.83 | 0.72-0.94 | 1.43 | 1.21 |
| **QDC** | 1.68 | 1.39-2.03 | 1.92 | 1.64 |
| **YADC** | 1.06 | 0.84-1.35 | 1.20 | 1.00 |
| **YIDC** | 1.03 | 0.81-1.32 | 1.14 | 1.00 |
| **PDC** | 0.89 | 0.67-1.19 | 1.31 | 1.00 |
| **DHC** | 1.24 | 0.98-1.58 | 1.47 | 1.00 |
| **BSC** | 1.13 | 0.87-1.45 | 1.32 | 1.00 |
| **QSC** | 2.53 | 2.00-3.23 | 2.56 | 2.18 |
| **ISC** | 0.90 | 0.69-1.19 | 1.29 | 1.00 |

^1^Conditional on measured covariates, the E-value is the minimum strength of association that an unmeasured confounder must have with the exposure and outcome to explain away the observed association.

Abbreviations: OR = Odds Ratio, CI = Confidence Interval; BC: balanced constitution; QDC: qi-deficiency constitution; YADC: yang-deficiency constitution; YIDC: yin-deficiency constitution; PDC: phlegm-dampness constitution; DHC: dampness-heat constitution; BSC: blood-stasis constitution; QSC: qi-stagnation constitution; ISC: inherited special constitution.

**Table S2: Conditional Multivariate Logistic regression analyzes after conducting the propensity score matching^1^ (N = 3872)**

| **Characteristic** | **Multivariable** | | |
| --- | --- | --- | --- |
|  | **OR** | **95% CI** | ***p*-value** |
| **BC** |  |  |  |
| *No* | — | — |  |
| *Yes* | 0.90 | 0.81, 1.01 | **0.065** |
| **QDC** |  |  |  |
| *No* | — | — |  |
| *Yes* | 1.20 | 1.05, 1.38 | **0.009** |
| **YADC** |  |  |  |
| *No* | — | — |  |
| *Yes* | 0.98 | 0.84, 1.15 | 0.817 |
| **YIDC** |  |  |  |
| *No* | — | — |  |
| *Yes* | 1.02 | 0.87, 1.20 | 0.812 |
| **PDC** |  |  |  |
| *No* | — | — |  |
| *Yes* | 0.95 | 0.79, 1.14 | 0.568 |
| **DHC** |  |  |  |
| *No* | — | — |  |
| *Yes* | 1.12 | 0.96, 1.31 | 0.159 |
| **BSC** |  |  |  |
| *No* | — | — |  |
| *Yes* | 1.02 | 0.86, 1.20 | 0.835 |
| **QSC** |  |  |  |
| *No* | — | — |  |
| *Yes* | 1.37 | 1.18, 1.60 | **<0.001** |
| **ISC** |  |  |  |
| *No* | — | — |  |
| *Yes* | 1.01 | 0.86, 1.20 | 0.885 |

^1^Propensity score matching by matching participants pairs in a ratio of 1:1 by age, gender, race, health status, whether only child and whether from a single-parent family

Abbreviations: OR = Odds Ratio; CI = Confidence Interval; BC: balanced constitution; QDC: qi-deficiency constitution; YADC: yang-deficiency constitution; YIDC: yin-deficiency constitution; PDC: phlegm-dampness constitution; DHC: dampness-heat constitution; BSC: blood-stasis constitution; QSC: qi-stagnation constitution; ISC: inherited special constitution.

**Table S3. Subgroup and Interaction Analyses of the Association Between TCMC Types and Depression**

| **Subgroup** | **OR(95%CI)** | **P for interaction** |
| --- | --- | --- |
| **BC** | 0.83 (0.73,0.95) |  |
| Gender |  | 0.358 |
| Female | 0.91 (0.72,1.16) |  |
| Male | 0.83 (0.71,0.97) |  |
| Age |  | 0.64 |
| <=18 | 0.85 (0.69,1.05) |  |
| >18 | 0.80 (0.68,0.94) |  |
| **YADC** | 1.06 (0.84,1.33) |  |
| Gender |  | 0.212 |
| Female | 0.94 (0.69,1.29) |  |
| Male | 1.20 (0.84,1.71) |  |
| Age |  | 0.265 |
| <=18 | 1.08 (0.74,1.58) |  |
| >18 | 1.07 (0.79,1.44) |  |
| **YIDC** | 1.05 (0.82,1.33) |  |
| Gender |  | 0.997 |
| Female | 1.18 (0.83,1.66) |  |
| Male | 0.92 (0.65,1.30) |  |
| Age |  | 0.433 |
| <=18 | 0.97 (0.64,1.48) |  |
| >18 | 1.06 (0.79,1.44) |  |
| **PDC** | 0.89 (0.67,1.18) |  |
| Gender |  | 0.297 |
| Female | 0.80 (0.54,1.20) |  |
| Male | 0.97 (0.65,1.47) |  |
| Age |  | 0.193 |
| <=18 | 0.97 (0.60,1.57) |  |
| >18 | 0.84 (0.59,1.20) |  |
| **DHC** | 1.22 (0.96,1.55) |  |
| Gender |  | 0.117 |
| Female | 1.04 (0.73,1.47) |  |
| Male | 1.43 (1.03,1.99) |  |
| Age |  | 0.753 |
| <=18 | 0.97 (0.65,1.43) |  |
| >18 | 1.46 (1.07,1.99) |  |
| **BSC** | 1.15 (0.89,1.48) |  |
| Gender |  | 0.469 |
| Female | 1.11 (0.80,1.53) |  |
| Male | 1.20 (0.79,1.83) |  |
| Age |  | 0.052 |
| <=18 | 1.41 (0.92,2.15) |  |
| >18 | 1.01 (0.74,1.39) |  |
| **QSC** | 2.61 (2.06,3.31) |  |
| Gender |  | 0.231 |
| Female | 3.38 (2.41,4.73) |  |
| Male | 1.95 (1.39,2.74) |  |
| Age |  | 0.026 |
| <=18 | 3.31 (2.18,5.02) |  |
| >18 | 2.27 (1.70,3.05) |  |
| **ISC** | 0.90 (0.68,1.18) |  |
| Gender |  | 0.362 |
| Female | 0.82 (0.56,1.21) |  |
| Male | 0.96 (0.65,1.41) |  |
| Age |  | 0.378 |
| <=18 | 0.85 (0.54,1.35) |  |
| >18 | 0.91 (0.65,1.27) |  |
| **QDC** | 1.69 (1.40,2.05) |  |
| Gender |  | 0.688 |
| Female | 1.70 (1.28,2.26) |  |
| Male | 1.72 (1.33,2.22) |  |
| Age |  | 0.055 |
| <=18 | 1.90 (1.39,2.60) |  |
| >18 | 1.57 (1.23,1.99) |  |

OR = Odds Ratio, CI = Confidence Interval; BC: balanced constitution; QDC: qi-deficiency constitution; YADC: yang-deficiency constitution; YIDC: yin-deficiency constitution; PDC: phlegm-dampness constitution; DHC: dampness-heat constitution; BSC: blood-stasis constitution; QSC: qi-stagnation constitution; ISC: inherited special constitution.

**Table S4: Multivariate Logistic regression analyzes in students aged 14 to 19 years (n=5081)**

| **Characteristic** | **Multivariable** | | |
| --- | --- | --- | --- |
|  | **OR** | **95% CI** | ***p*-value** |
| **Age** | 0.78 | 0.74, 0.83 | **<0.001** |
| **Gender** |  |  |  |
| *Female* | — | — |  |
| *Male* | 0.89 | 0.78, 1.02 | 0.094 |
| **From a one-child family** |  |  |  |
| *No* | — | — |  |
| *Yes* | 0.76 | 0.65, 0.88 | **<0.001** |
| **From a single-parent family** |  |  |  |
| *No* | — | — |  |
| *Yes* | 1.23 | 1.00, 1.50 | **0.049** |
| **BC** |  |  |  |
| *No* | — | — |  |
| *Yes* | 0.84 | 0.73, 0.97 | **0.016** |
| **QDC** |  |  |  |
| *No* | — | — |  |
| *Yes* | 1.58 | 1.29, 1.95 | **<0.001** |
| **YADC** |  |  |  |
| *No* | — | — |  |
| *Yes* | 0.99 | 0.76, 1.28 | 0.931 |
| **YIDC** |  |  |  |
| *No* | — | — |  |
| *Yes* | 1.10 | 0.84, 1.44 | 0.485 |
| **PDC** |  |  |  |
| *No* | — | — |  |
| *Yes* | 0.86 | 0.63, 1.18 | 0.346 |
| **DHC** |  |  |  |
| *No* | — | — |  |
| *Yes* | 1.25 | 0.96, 1.63 | 0.096 |
| **BSC** |  |  |  |
| *No* | — | — |  |
| *Yes* | 1.08 | 0.82, 1.43 | 0.582 |
| **QSC** |  |  |  |
| *No* | — | — |  |
| *Yes* | 2.69 | 2.08, 3.49 | **<0.001** |
| **ISC** |  |  |  |
| *No* | — | — |  |
| *Yes* | 0.90 | 0.67, 1.21 | 0.473 |

OR = Odds Ratio, CI = Confidence Interval; BC: balanced constitution; QDC: qi-deficiency constitution; YADC: yang-deficiency constitution; YIDC: yin-deficiency constitution; PDC: phlegm-dampness constitution; DHC: dampness-heat constitution; BSC: blood-stasis constitution; QSC: qi-stagnation constitution; ISC: inherited special constitution.

**Table S5: Univariate and multivariate Logistic regression analyzes in students of all ages (n=5994)**

|  | **Univariable** | | | | **Multivariable** | | |
| --- | --- | --- | --- | --- | --- | --- | --- |
| **Characteristic** | **N** | **OR** | **95% CI** | **p-value** | **OR** | **95% CI** | **p-value** |
| **Age** | 5,994 | 0.87 | 0.84, 0.91 | **<0.001** | 0.87 | 0.84, 0.91 | **<0.001** |
| **Gender** | 5,994 |  |  |  |  |  |  |
| *Female* |  | — | — |  | — | — |  |
| *Male* |  | 0.66 | 0.59, 0.74 | **<0.001** | 0.93 | 0.82, 1.06 | 0.284 |
| **From a one-child family** | 5,994 |  |  |  |  |  |  |
| *No* |  | — | — |  | — | — |  |
| *Yes* |  | 0.80 | 0.71, 0.92 | **0.001** | 0.77 | 0.67, 0.89 | **<0.001** |
| **From a single-parent family** | 5,994 |  |  |  |  |  |  |
| *No* |  | — | — |  | — | — |  |
| *Yes* |  | 1.27 | 1.07, 1.51 | **0.006** | 1.27 | 1.06, 1.53 | **0.012** |
| **Race** | 5,994 |  |  |  |  |  |  |
| *ethnic minorities* |  | — | — |  |  |  |  |
| *Han ethnicity* |  | 1.03 | 0.82, 1.28 | 0.827 |  |  |  |
| **BC** | 5,994 |  |  |  |  |  |  |
| *No* |  | — | — |  | — | — |  |
| *Yes* |  | 0.49 | 0.44, 0.55 | **<0.001** | 0.83 | 0.72, 0.94 | **0.004** |
| **QDC** | 5,994 |  |  |  |  |  |  |
| *No* |  | — | — |  | — | — |  |
| *Yes* |  | 3.20 | 2.78, 3.69 | **<0.001** | 1.69 | 1.40, 2.04 | **<0.001** |
| **YADC** | 5,994 |  |  |  |  |  |  |
| *No* |  | — | — |  | — | — |  |
| *Yes* |  | 2.74 | 2.29, 3.31 | **<0.001** | 1.07 | 0.84, 1.35 | 0.593 |
| **YIDC** | 5,994 |  |  |  |  |  |  |
| *No* |  | — | — |  | — | — |  |
| *Yes* |  | 2.81 | 2.36, 3.37 | **<0.001** | 1.04 | 0.81, 1.32 | 0.782 |
| **PDC** | 5,994 |  |  |  |  |  |  |
| *No* |  | — | — |  | — | — |  |
| *Yes* |  | 2.97 | 2.44, 3.63 | **<0.001** | 0.89 | 0.67, 1.18 | 0.419 |
| **DHC** | 5,994 |  |  |  |  |  |  |
| *No* |  | — | — |  | — | — |  |
| *Yes* |  | 2.76 | 2.31, 3.31 | **<0.001** | 1.24 | 0.98, 1.58 | 0.075 |
| **BSC** | 5,994 |  |  |  |  |  |  |
| *No* |  | — | — |  | — | — |  |
| *Yes* |  | 3.09 | 2.57, 3.74 | **<0.001** | 1.14 | 0.88, 1.47 | 0.318 |
| **QSC** | 5,994 |  |  |  |  |  |  |
| *No* |  | — | — |  | — | — |  |
| *Yes* |  | 4.56 | 3.80, 5.52 | **<0.001** | 2.53 | 2.00, 3.21 | **<0.001** |
| **ISC** | 5,994 |  |  |  |  |  |  |
| *No* |  | — | — |  | — | — |  |
| *Yes* |  | 2.56 | 2.06, 3.21 | **<0.001** | 0.90 | 0.69, 1.19 | 0.471 |

Abbreviations: OR = Odds Ratio; CI = Confidence Interval; BC: balanced constitution; QDC: qi-deficiency constitution; YADC: yang-deficiency constitution; YIDC: yin-deficiency constitution; PDC: phlegm-dampness constitution; DHC: dampness-heat constitution; BSC: blood-stasis constitution; QSC: qi-stagnation constitution; ISC: inherited special constitution.

**Table S6: Univariate and multivariate logistic regression analyses for clinically significant depression (SDS ≥ 60) (N = 5955)**

| **Characteristic** | **Univariable** | | | **Multivariable** | | | |
| --- | --- | --- | --- | --- | --- | --- | --- |
|  | **OR** | **95% CI** | **p-value** | **OR** | **95% CI**^1^ | **p-value** | **VIF** |
| **Age** | 0.98 | 0.94, 1.01 | 0.202 |  |  |  |  |
| **Gender** |  |  |  |  |  |  | 1.1 |
| *Female* | — | — |  | — | — |  |  |
| *Male* | 0.76 | 0.68, 0.84 | **<0.001** | 0.99 | 0.88, 1.11 | 0.844 |  |
| **From a one-child family** |  |  |  |  |  |  | 1.1 |
| *No* | — | — |  | — | — |  |  |
| *Yes* | 0.89 | 0.78, 1.01 | **0.075** | 0.86 | 0.74, 0.99 | **0.034** |  |
| **From a single-parent family** |  |  |  |  |  |  | 1.1 |
| *No* | — | — |  | — | — |  |  |
| *Yes* | 1.35 | 1.16, 1.58 | **<0.001** | 1.32 | 1.11, 1.56 | **0.001** |  |
| **Race** |  |  |  |  |  |  |  |
| *Ethnic minorities* | — | — |  |  |  |  |  |
| *Han ethnicity* | 1.01 | 0.81, 1.26 | 0.957 |  |  |  |  |
| **BC** |  |  |  |  |  |  | 1.5 |
| *No* | — | — |  | — | — |  |  |
| *Yes* | 0.62 | 0.55, 0.69 | **<0.001** | 1.1 | 0.96, 1.26 | 0.155 |  |
| **QDC** |  |  |  |  |  |  | 2 |
| *No* | — | — |  | — | — |  |  |
| *Yes* | 2.57 | 2.29, 2.89 | **<0.001** | 1.6 | 1.35, 1.89 | **<0.001** |  |
| **YADC** |  |  |  |  |  |  | 1.7 |
| *No* | — | — |  | — | — |  |  |
| *Yes* | 2.34 | 2.03, 2.71 | **<0.001** | 1.09 | 0.90, 1.32 | 0.379 |  |
| **YIDC** |  |  |  |  |  |  | 2 |
| *No* | — | — |  | — | — |  |  |
| *Yes* | 2.35 | 2.05, 2.70 | **<0.001** | 1.03 | 0.84, 1.26 | 0.805 |  |
| **PDC** |  |  |  |  |  |  | 2.2 |
| *No* | — | — |  | — | — |  |  |
| *Yes* | 2.58 | 2.21, 3.00 | **<0.001** | 1.03 | 0.82, 1.30 | 0.799 |  |
| **DHC** |  |  |  |  |  |  | 1.9 |
| *No* | — | — |  | — | — |  |  |
| *Yes* | 2.24 | 1.94, 2.58 | **<0.001** | 1.09 | 0.89, 1.33 | 0.42 |  |
| **BSC** |  |  |  |  |  |  | 2 |
| *No* | — | — |  | — | — |  |  |
| *Yes* | 2.63 | 2.28, 3.04 | **<0.001** | 1.24 | 1.01, 1.52 | **0.042** |  |
| **QSC** |  |  |  |  |  |  | 1.9 |
| *No* | — | — |  | — | — |  |  |
| *Yes* | 3.22 | 2.82, 3.67 | **<0.001** | 2.05 | 1.71, 2.47 | **<0.001** |  |
| **ISC** |  |  |  |  |  |  | 1.5 |
| *No* | — | — |  | — | — |  |  |
| *Yes* | 2.19 | 1.84, 2.60 | **<0.001** | 0.91 | 0.73, 1.13 | 0.389 |  |
| Abbreviations: OR = Odds Ratio; CI = Confidence Interval; VIF = Variance Inflation Factor; BC: balanced constitution; QDC: qi-deficiency constitution; YADC: yang-deficiency constitution; YIDC: yin-deficiency constitution; PDC: phlegm-dampness constitution; DHC: dampness-heat constitution; BSC: blood-stasis constitution; QSC: qi-stagnation constitution; ISC: inherited special constitution. | | | | | | | |

**
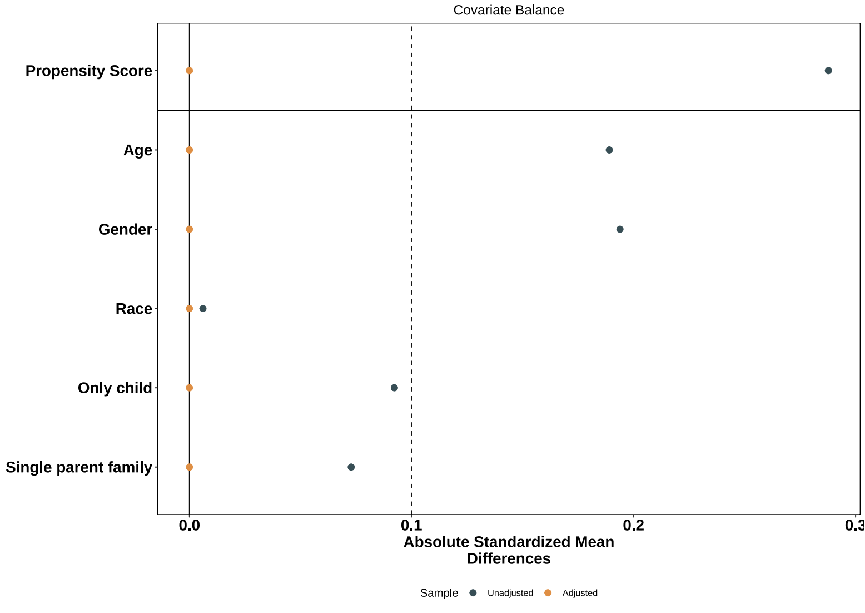
**

**Figure S1: Change of standardized mean difference before and after propensity score matching**

**
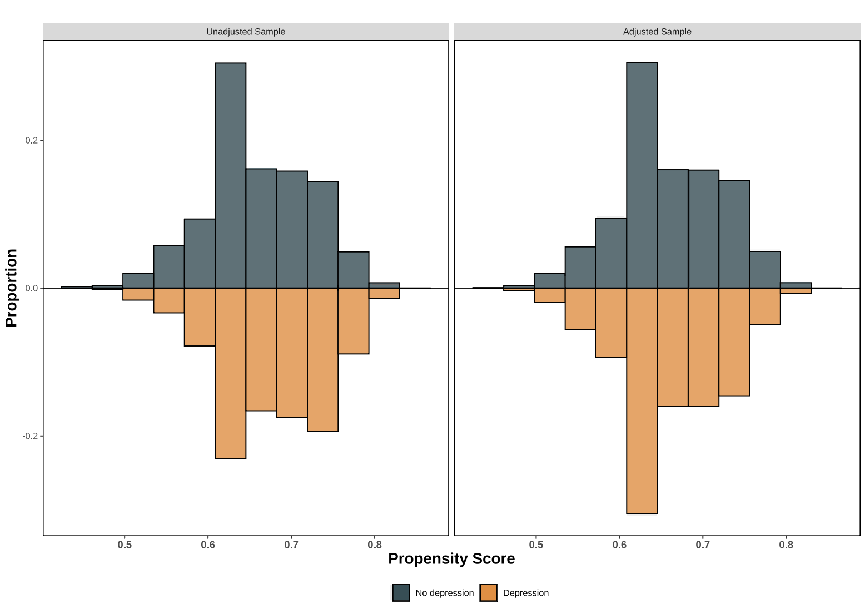
**

**Figure S2: Density distribution of propensity score values in the depressed and non-depressed groups before and after matching**
